# Exploring the Effectiveness of Biological Therapy in Patients with Psoriasis: Body Image and Quality of Life

**DOI:** 10.3390/medicina60010160

**Published:** 2024-01-15

**Authors:** Chia-Lien Wu, Ya-Ching Chang, Wen-Teng Yao, Tsay-I Chiang

**Affiliations:** 1 Department of Dermatology, Chang Gung Memorial Hospital, Linkou, Taoyuan 333, Taiwan; claire16948@cgmh.org.tw (C.-L.W.); ycchang@cgmh.org.tw (Y.-C.C.); 2 Division of Plastic Surgery, Department of Surgery, Mackay Memorial Hospital, Taipei 104, Taiwan; hippo.4825@mmh.org.tw; 3 Department of Materials Science and Engineering, National Taiwan University, Taipei 106, Taiwan; 4 Department of Nursing, Hungkuang University, Taichung 403, Taiwan

**Keywords:** psoriasis, biologics, body image, quality of life

## Abstract

*Background and Objectives*: Psoriasis is a chronic, long-term, incurable skin inflammatory disease characterized by the excessive proliferation of epidermal keratinocytes, dilation of blood vessels, thickening of the skin, and the formation of visible red patches of variable sizes. The impact on patients differs with the severity of the disease, leading to physiological discomfort and psychological distress, which significantly affect the quality of life. The etiology of psoriasis is not completely clear, but immune cells, including type 1 and type 17 cytokine-producing cells modulated by regulatory T cells (Tregs), play a critical role in driving the disease pathogenesis. With the ability to specifically target inflammatory markers, biologics can efficiently inhibit the spread of inflammation to achieve therapeutic effects. The goal was to explore the changes in body image and quality of life in psoriasis patients undertaking therapies with biologic agents. *Materials and Methods*: This study employed a quasi-experimental, single-sample, pretest–posttest design. Forty-four psoriasis patients were recruited from the dermatology outpatient clinics at two medical centers in northern Taiwan. A structured questionnaire, including demographic information, the Body Image Scale (BIS), and the Dermatology Life Quality Index (DLQI), was used as a research tool. Questionnaire assessments were conducted both before and three months after the biologic agent intervention. Statistical analyses were performed using SPSS version 22.0. *Results*: Our results indicated a significant difference in body image between psoriasis patients before and after intervention with biologic agents. In addition, overall quality of life (QoL) also showed significant improvements before and after biologic agent intervention. There was a positive correlation between body image and quality of life in psoriasis patients. *Conclusions*: The treatment for psoriasis has evolved rapidly in recent years, and biologic agents have proven to be effective therapies to improve the quality of life for psoriasis patients. Our study suggests that health-related education and psychological support can further benefit psoriasis patients to willingly and positively undertake treatment and therefore improve their positive body image and quality of life.

## 1. Introduction

Psoriasis is a chronic, recurrent, systemic inflammatory skin disease primarily characterized by the excessive proliferation of epidermal keratinocytes, leading to blood vessel dilation, skin thickening, and the formation of red patches of variable sizes covered with multiple layers of silver white scales. Psoriasis lesions typically occur on the surfaces of the knees, elbows, scalp, and trunk. It is an autoimmune disease affecting individuals of all ages [1,2] and can be classified into two types based on the average age of disease onset. Type I, early-onset psoriasis, occurs before the age of 40, with a peak onset age between 16 and 22 years, and accounts for 70% of all psoriasis cases. Type II, late-onset psoriasis, develops after the age of 40 with a peak onset age between 57 and 60 years [3]. The probability of occurrence is equal between males and females [4].

The global prevalence of psoriasis ranges from 0.09% to 11.43%, affecting approximately 125 million people worldwide [5]. The prevalence of psoriasis is approximately 0.235% in Taiwan [6,7], significantly lower than that in Caucasians (2–11%), African Americans (1.3%), Indians (0.5–1.5%), Malaysians (4–5.5%), Japanese (0.29–1.18%), and Koreans (0.44–0.45%). The incidence rates in the Taiwan population for males and females are 0.23% and 0.16%, respectively, with a noticeable increase in the incidence rate in males aged 30 and above [8]. According to the Taiwan National Health Insurance (NHI) database, about 50,000 to 100,000 people take medical treatment for psoriasis each year [9]. Additionally, the number of new cases is increasing at a rate of at least 3000 patients annually [10]. Although psoriasis does not pose a threat to life, the impact on patients varies with the severity of the disease. It ranges from localized skin scaling to the development of widespread psoriasis or concurrent psoriatic arthritis [11].

The etiology of psoriasis is not fully elucidated; both exogenous stimuli and endogenous factors can induce the disease. The disease is a result of a network of diversified cells including immune cells and hyperactive keratinocytes [12]. Type 1 and type 17 cytokine-producing cells modulated by Tregs play a critical role in driving the disease pathogenesis. Tregs play a central role in immune homeostasis by suppressing immune responses. In psoriasis, Tregs are impaired in their suppressive function, leading to an imbalanced T-helper 17/Treg. The secretion of cytokines generates a chronic inflammatory environment, and the crosstalk between these cells contributes to the pathological phenotype.

The treatment of psoriasis depends on the severity of the disease. For mild psoriasis, topical ointments are initially used. If the affected area is extensive, consideration should be given to systemic UV light therapy. Systemic oral medications may be added if UV light therapy combined with topical ointments fails to effectively control the condition. Biologic therapy should be given when the above treatments are not successful in controlling the disease [13]. Biologics inhibit the spread of inflammation by specifically targeting inflammatory markers to achieve therapeutic effects [13]. Compared to traditional topical or oral medications, biologics have the advantages of superior efficacy and fewer hepatorenal toxic side effects. The first psoriasis biologics is Amevive approved by the United States Food and Drug Administration in 2003. Biologics are categorized based on their mechanisms of action targeting tumor necrosis factor-α (TNF-α), interleukin 12/23 (IL12/23), IL17, or IL23 [13]. Currently available biologics for psoriasis treatment in Taiwan are etanercept, adalimumab, ustekinumab, secukinumab, ixekizumab, brodalumab, guselkumab, and risankizumab [13].

The skin, being the largest organ in the body, serves as the major representation of external appearance. It plays a critical role in interacting with the world and is a key element in maintaining human psychological states and psychological adaptation. It also represents an important element in body image and the establishment of self-esteem. Body image is a multidimensional experience involving thoughts, emotions, sensations, behaviors, self-perception, and perceptions of the body, particularly those related to outward appearance [14]. Most of an individual’s perception of his or her body image depends on societal feedback. The health of the skin is closely intertwined with the reciprocal influence of both the physical and psychological aspects. Losing healthy skin can impair one’s psychosocial functioning and lead to a negative body image [10,15]. Skin imperfections resulting from psoriasis can lead to emotional, social, and economic difficulties, contributing to a decline in the quality of life [16].

Although psoriasis normally does not affect survival, it certainly causes negative effects on individual’s daily life in many ways, including body image and quality of life. With the development of biologic therapies and diverse treatment options for psoriasis, the possibility of not leaving skin lesions after treatment has become a reality. Since there are limited studies focusing on the body image and quality of life regarding biologic therapy, our aim was to understand the correlation and effectiveness of body image and quality of life in psoriasis patients before and after intervention with biologic therapy. Hopefully, our study can provide healthcare professionals with information to assist psoriasis patients in actively pursuing treatments and overcoming the challenges posed by psoriasis.

## 2. Materials and Methods

### 2.1. Study Design

This study is a quasi-experimental, single-sample, longitudinal research design conducted using a questionnaire survey method. The questionnaire contains three parts: individual sociodemographic information, the BIS and the DLQI. Psoriasis patients from the dermatology outpatient departments of two medical centers in the northern region of Taiwan were selected as the study subjects. The study participants recruited for our research must meet the following criteria:

1. Individuals diagnosed with psoriasis based on diagnostic criteria and confirmed by dermatologists or rheumatologists.
2. Participants undergoing biologic treatment, including the following:
  (1) First-time recipients of biologic treatment.
  (2) Individuals experiencing a psoriasis recurrence after discontinuing biologic treatment and subsequently resuming it.
  (3) Those transitioning to a different type of biologic treatment.
3. Age 20 years or older, mentally alert, and capable of communicating in Mandarin or Taiwanese.
4. Willingness to participate in the study and the ability to sign the informed consent form.

**Figure 1:**
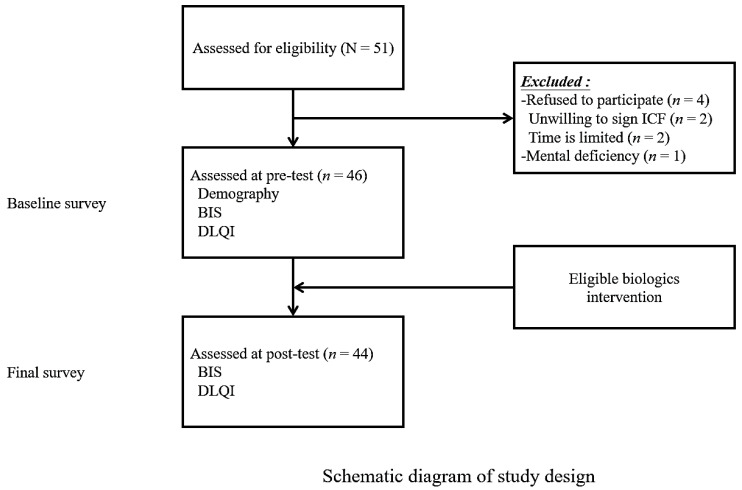
Schematic diagram of study design. BIS, Body Image Scale. DLQI, Dermatology Life Quality Index.

Our study employs purposive sampling and utilizes a structured questionnaire as the research tool for data collection. The collected data will be exclusively used for research purposes and will not be employed for any other questionnaire-related activities. However, with privacy considerations in mind, the data collected for this study does not include information about patients’ medication history or the specific biological drugs administered. The questionnaire was administered at two time points. The baseline survey was conducted before the intervention with biologic therapy, and the final survey was conducted three months after biologic therapy. The sample size for this study was estimated using G Power 3.1.9.4 statistical software, with a moderate effect size estimate of 0.5, alpha of 0.05, and power of 0.8, resulting in a sample size of 34. Considering a 30% attrition rate and dropouts, 50 participants were planned to be recruited. In the actual recruitment, 51 psoriasis patients were expected to undertake biologic intervention during the study. After screening, a total of 46 eligible study subjects were recruited. All participants signed an informed consent form and completed the baseline questionnaire. However, two participants did not complete the second follow-up questionnaire. Therefore, the final subject number was 44. A schematic diagram of study design is shown in Figure 1.

### 2.2. Sociodemographic Data

This includes age, gender, education level, occupation, marital status, religious beliefs, socioeconomic status, chronic diseases, major locations of psoriasis lesions, smoking, alcohol consumption, regular exercise, body mass index (BMI), and severity of psoriasis (PASI).

### 2.3. Body Image Scale, BIS

The body image assessment covers emotions, behaviors, and cognition. The Body Image Scale consists of a total of 10 questions, scored on a 4-point Likert scale. The scores for each item are summed to obtain a total score ranging from 0 to 30, where higher scores indicate a more negative body image perception.

### 2.4. Dermatology Life Quality Index, DLQI

The Dermatology Life Quality Index is the most used measurement tool for assessing the quality of life of dermatology patients. This tool was developed by scholars Finlay and Khan in 1994. The scale is designed to measure the degree of distress caused by skin problems in patients’ daily lives over the past week. The questions are categorized into six domains, including Disease Symptoms, Daily Activities, Leisure Activities, Work and Study, Interpersonal Relationships, and Treatment. Respondents provide scores using a 4-point Likert scale. Higher total scores indicate a greater effect on quality of life.

### 2.5. Data Processing and Statistical Analysis

After collecting the data, statistical analysis was conducted using the SPSS v22.0 (Statistical Package for the Social Sciences, New York, NY, USA). Analytical techniques included frequency distribution, mean, standard deviation (SD), percentage, independent samples *t*-test, paired sample *t*-test, one-way analysis of variance (ANOVA), and Pearson product-moment correlation were used to explore relationships within the data.

The sociodemographic variables were analyzed using frequency distribution, mean, standard deviation, and percentages. The paired sample *t*-test was used to compare the differences in body image and the quality of life of psoriasis patients before and after intervention with biologic therapy. The independent sample *t*-test, one-way ANOVA, Pearson product-moment correlation analysis, and linear regression analysis were employed to explore the relationships among the demographic characteristics, body image, and quality of life of psoriasis patients.

## 3. Results

### 3.1. Sociodemographic Analysis of the Study Population

The study comprised 44 subjects, including 33 men and 11 women, with a mean age of 47.1 years. More than 50% of our respondents were married (61%), and one-fourth of the subjects were single. Half of the patients had an education level of college or above. About 70% of the patients had a middle or middle-to-high socioeconomic status. The study group was composed of 34% smokers, 4 former smokers and 57% non-smokers. More than half of the patients did not consume alcohol (75%). About half of the patients had religious beliefs, and more than half of the subjects suffered from chronic diseases (57%). Seventy-five percent of respondents had a non-healthy body weight (BMI < 18.5 and BMI ≥ 24). About half of the patients exercised regularly (55%). More than three-quarter of the respondents had moderate to severe PASI, and the major sites of psoriasis lesions were located on the limbs (36%) followed by trunk (27%), face (18%), and scalp (18%). The general characteristics of the study participants are illustrated in Table 1.

### 3.2. Psoriasis Poses a Negative Impact on Body Image

Body image is a multidimensional concept containing both positive and negative features. Interest in body image has increased in recent years. We assessed whether the perceptions and cognitions of each subject’s body image was affected by different sociodemographic variables (Table 2). Our results demonstrate that the perceptions and cognitions of each subject’s body image were affected by education level (*t* = 3.652, *p* = 0.001). The impact on body image is higher among individuals with an education level of high school or below than those of with an education level of college and above. On the other hand, there are no statistical differences regarding gender (*t* = −1.233, *p* = 0.225), chronic diseases (*t* = −1.353, *p* = 0.183), BMI groups (*t* = 0.231, *p* = 0.818), regular exercise (t = −0.701, *p* = 0.487), PASI severity (*t* = −0.728, *p* = 0.471), age (F = 0.466, *p* = 0.631), marital status (F = 1.931, *p* = 0.158), income (F = 1.525, *p* = 0.223), major locations of psoriasis lesions (F = 0.271, *p* = 0.846), smoking (F = 0.620, *p* = 0.543) and alcohol consumption (F = 1.298, *p* = 0.284) (Table 2).

Although there were statistically significant differences in socioeconomic status (F = 3.316, *p* = 0.046) and religious beliefs (F = 3.891, *p* = 0.028), post hoc comparisons using the Scheffé method revealed no statistical differences among socioeconomic status groups, while respondents who cited Buddhist religious beliefs experienced a higher impact on their body image compared to non-religious respondents (Table 2).

### 3.3. Differential Impact of Sociodemographic Variables on Quality of Life

We next examined the impact of sociodemographic variables on six aspects, including Disease Symptoms, Daily Activities, Leisure Activities, Work and Study, Interpersonal Relationships, and Treatment under the domain of quality of life (Table 3 and Supplementary Tables S1–S13). We observed a higher impact in Work and Study for females compared to males (*t* = −2.130, *p* = 0.039). Those with an education level of high school or below experience a greater impact than those with a college education or above in Disease Symptoms (*t* = 3.984, *p* = 0.000), Daily Activities (*t* = 4.045, *p* = 0.000), Leisure Activities (*t* = 3.258, *p* = 0.002), Interpersonal Relationships (*t* = 3.055, *p* = 0.004), and Treatment (*t* = 3.584, *p* = 0.001). Regarding PASI severity, only Treatment was observed to be significantly different (*t* = −2.132, *p* = 0.039). Patients with moderate to severe psoriasis experience a higher impact in Treatment compared to patients with mild psoriasis.

In addition, respondents with a middle socioeconomic status were more affected than those with a middle-to-high socioeconomic status in Disease Symptoms (*t* = 4.621, *p* = 0.015) and Interpersonal Relationships (*t* = 3.564, *p* = 0.037). Regarding religious beliefs, only Interpersonal Relationships showed a significant difference (F = 5.956, *p* = 0.005). Post hoc comparisons demonstrate that Buddhists experienced a higher impact compared to those who identified as non-religious. Concerning the major locations of psoriasis lesions, both Work and Study (F = 3.262, *p* = 0.031) and Treatment (F = 3.176, *p* = 0.034) were statistically different. This finding indicates that under the quality of life domain, the Work and Study and Treatment variables may vary due to differences in psoriasis lesion sites. However, further analysis with Scheffe post hoc comparisons demonstrates that there were no significant differences among Work and Study groups. Interestingly, the demographic variables of chronic diseases, BMI group, regular exercise, age, marital status, income, smoking, and alcohol consumption showed no differences in all the six aspects under the domain of quality of life.

### 3.4. Intervention with Biologic Agents Significantly Improves the Overall Body Image and Quality of Life

When body perception exhibits a negative body image, it not only affects mental health but also influences treatment adherence and outcomes. The treatment landscape for psoriasis has greatly evolved, and the application of biologic agents has proven to be an effective way to treat psoriasis. Therefore, we examined whether there were differences in body image before and after biologic therapy intervention. The results show a *t*-value of 14.205 (*p* < 0.001), indicating a significant difference in body image after biologic therapy intervention (Table 4). Based on the statistical mean results, it is evident that the scores in the pre-intervention assessment were higher than those in the post-intervention assessment (Table 4).

In addition to body image, we also explored whether there were differences in the six aspects under the quality of life domain, and the results demonstrate that all variables reach statistical significance (Table 5). According to the mean results, the scores in the pre-intervention assessment were higher than those in the post-intervention assessment for all the six aspects, indicating that biologic therapy intervention effectively improves the quality of life for psoriasis patients (Table 5).

### 3.5. Positive Correlation between Body Image and Quality of Life

Our results reveal that both body image and quality of life of psoriasis patients are affected by the disease. We next used Pearson correlation analysis to examine the linear correlation between the two variables. The correlation coefficient value between pre-test body image and pre-test quality of life was r(44) = 0.726 (*p* < 0.001, Table 6). The correlation coefficient value between post-test body image and post-test quality of life was r(44) = 0.690 (*p* < 0.001, Table 7). It Is evident that there is a highly positive correlation between body image and quality of life.

We further used linear regression analysis to understand the predictive impact of body image on quality of life. The standardized regression coefficient (Beta) for body image was 0.726, and the non-standardized coefficient (B) was 0.888 (Table 8). For every 1-point increase in the body image score, the total score of quality of life increased by 0.888 points, and the difference was statistically significant (*p* < 0.001, Table 8). Body image was able to explain 51.6% of the variance in quality of life (Adjusted *R*^2^ = 0.516), indicating a significant impact of body image on quality of life.

## 4. Discussion

### 4.1. Psoriasis and Body Image

Negative body image arises when there is a significant difference between an individual’s perceived real body image and his or her desired body image. Our study found that although psoriasis patients’ gender, age, marital status, socioeconomic status, income, presence of chronic diseases, exercise patterns, BMI, PASI severity, and smoking or drinking habits did not show a significant impact on body image, the average score at baseline was high at 18.89. Noticeably, 3 patients scored the maximum of 30 points, indicating a substantial impact of psoriasis on patients’ body image. A global study comprising 8338 moderate-to-severe psoriasis patients from 31 countries revealed that 84% patients experienced discrimination or humiliation due to psoriasis [17]. It can potentially cause social exclusion, especially when there is a need to expose one’s body. Appearance concerns, particularly visible skin conditions, may relate to any part of the body. In a study by Ho, skin defects in psoriasis patients were identified as the primary factor influencing body image [10].

Regarding education background, our results align with Nazik et al.’s findings, showing that participants with a higher education level had better body image perceptions [18]. In our study, marital status, income, and socioeconomic status showed no significant impact on body image. This is slightly different from the findings of Rzeszutek et al., who reported that psoriasis patients had lower life satisfaction, a more negative body image, and poorer social-psychological resources compared to healthy controls [19]. The different results may be due to the research being based on the division of overall resources (e.g., mental resources, power, and prestige), rather than merely comparing a single variable (e.g., religious beliefs, socioeconomic status). As for religious beliefs, those who identified as Buddhist showed a greater impact on body image than those who identified as non-religious. Consequently, it might be essential to consider religious beliefs as part of patient care to avoid underestimating psychological issues and the severity of the disease.

### 4.2. Psoriasis and QoL

Our study found no significant impact on psoriasis patients based on age, marital status, income, presence of chronic diseases, exercise patterns, BMI, and smoking or drinking habits. Our results align with Yang’s study of 100 psoriasis patients in Taiwan [20]. Similarly, Lee et al. found no significant correlation among marital status, comorbidities, and health-related Quality of Life (HRQOL) [21]. While smoking or drinking habit did not directly impact patients’ quality of life in our finding, studies found that heavy smokers (more than 20 cigarettes per day) significantly increased the risk of severe psoriasis [22]. In addition, a study shows that 32.9% of drinkers had a higher risk of developing severe psoriasis compared to 67.1% of non-drinkers [23]. Further analysis needs to be performed to study the role of smoking and drinking habits in psoriasis patients’ QoL.

In terms of gender, although the average QoL score for men was 16.09 and that for women was 17.73, the difference did not reach statistical significance. However, women scored higher than men on all four aspects, including Disease Symptoms, Daily Activities, Study and Work, and Interpersonal Relationships. The impact on QoL was greater for women than for men, consistent with findings from the National Psoriasis Foundation [24]. This finding may be because female patients are more sensitive to the embarrassment caused by skin conditions, making them more prone to sensing external pressures compared to males [25]. Regarding education background, our findings align with the results of Feldman et al. and are consistent with the observations of Rzeszutek et al., indicating that life satisfaction was higher in the group with higher education [19,26]. Interestingly, Rzeszutek et al. found that residents in metropolitan areas with populations exceeding 500,000 reported higher life satisfaction than those in non-metropolitan areas [19]. The correlation between urban–rural differences and psoriasis-related QoL could be a future research consideration.

As for religious beliefs, our study found that individuals with Buddhist beliefs had a greater impact on the Interpersonal Relationships compared to those without religious beliefs. Noticeably, there is limited research on the correlation between religion and psoriasis patients’ QoL. Iranian scholars (Ghorbanibirgani et al., 2016) highlighted the significance of faith and religion in helping psoriasis patients cope with the disease, providing emotional support and contributing to a sense of peace [27]. Religious beliefs in Taiwan are closely intertwined with people’s lives, and whether there are deeper implications needs further investigation. In addition, the differences between Eastern and Western perspectives are also factors that need to be taken into consideration.

### 4.3. Psoriasis, Body Image, QoL, and Biologic Therapy

Before the intervention with biologic therapy, the average score for body image was 18.89, indicating that patients’ perception and cognition of body image were generally more negative (total scores >10 points). Notably, 4 individuals scored less than 10 points in the pre-test, indicating less disturbance in body image. Further exploring the reasons for lower scores revealed that patients experienced a numbing effect over time due to the chronic and recurrent nature of psoriasis. The average duration of illness for these 4 patients was greater than 10 years, and the long treatment period contributed to a sense of desensitization towards the disease. In Taiwan, a lower proportion of psoriasis patients expressed satisfaction with their treatment compared to global figures (35% vs. 56%). Dissatisfaction was commonly attributed to treatment failure and unsatisfactory skin appearance [28].

Compared to the pre-test, the post-test average score of 6.32 indicated that patients’ perception and cognition of body image shifted to be more positive after the intervention with biologic therapy. Studies have shown that biologic therapy enhances patients’ adherence to medication and treatment satisfaction [26,29,30]. In addition, patients with biologic therapy are more satisfied with their skin condition and have a better QoL than those receiving non-biologic therapies. Nevertheless, it is noteworthy that in each aspect of quality of life, the post-test results exhibit a relatively high standard deviation (Table 5). This suggests a wide range of individual responses among participants following the intervention. Several potential explanations for the elevated standard deviation in the post-test results are as follows:

1. Individual differences: Participants may have diverse responses to the intervention, with some individuals experiencing significant improvements in quality of life, while others may not experience the same level of change.
2. Small sample size: The recruitment of a relatively limited sample of 44 participants means that individual response variations can have a more pronounced impact on the standard deviation.
3. External influences: Uncontrollable factors, such as environmental impacts or additional treatments, could have influenced participants’ post-test scores outside the scope of the study.

Interestingly, an improvement in depression was observed during the process of biologic therapy. Whether this observation is a direct or an indirect result of biologic therapy needs further investigation [31]. In addition to body image, our result aligns with other studies indicating that biologic therapy also effectively improves psoriasis patients’ QoL. Mease and Menter confirmed that biologic therapy not only improves the disease but also enhances patients’ HRQOL [32]. A 10-year observational study in Sweden involving 583 psoriasis patients who had never used biologic therapy showed a significant improvement in PASI and DLQI 3–5 months after biologic therapy. Long-term use of biologic therapy demonstrated the sustained effectiveness in stabilizing the QoL [33].

Psoriasis is likened to a lifelong burden for patients. Therefore, treatment is an extremely important aspect regarding psoriasis patients’ QoL. For moderate to severe psoriasis, intervention with biologics can effectively improve patients’ perception and cognition of body image and their quality of life. Our results suggest that clinical assessments based solely on objective physiological values are insufficient to represent the overall severity of the disease. It is highly recommended that physicians need to assess patients’ inner world to address their psychological and social issues adequately. Collectively, to effectively improve the patients’ QoL, various dimensions, including psychological and social factors must be comprehensively considered.

### 4.4. Placebo Effect of Topical Therapy

Concerning psoriasis treatment, our study reveals a noteworthy distinction among patients with varying severity levels. Specifically, individuals with moderate to severe psoriasis appear to undergo a more noticeable impact during treatment compared to those with mild psoriasis. However, a paradoxical pattern emerges as we observe a lower proportion of patients expressing satisfaction with their psoriasis treatment. Psoriasis patients might not be as happy with their treatments for a few reasons. The condition itself is complicated, and those with more severe symptoms may expect more from their treatments. Also, psychological factors, such as thinking a treatment will work (placebo) or will not work well (nocebo), can play a part in how satisfied they feel.

Looking into how our minds affect treatments, a study on placebos and nocebos helps us understand the reasons behind lower satisfaction among people with psoriasis [34]. One plausible explanation for the reduced satisfaction could be the complex nature of psoriasis itself. Patients with moderate to severe psoriasis may experience a greater burden of symptoms, both physically and emotionally. They might expect treatments to work better and notice any problems more. Additionally, the chronic and relapsing nature of psoriasis may contribute to patients’ frustration and dissatisfaction. Moreover, the placebo effect plays a role in shaping patient satisfaction [34]. In the context of topical therapy for psoriasis, the placebo effect can be particularly significant. Simply applying a topical treatment, even if it lacks active therapeutic ingredients, can make some patients feel better because they believe it will help and want to see improvement. On the other hand, the nocebo effect could contribute to dissatisfaction. If someone believes a treatment will not work well, it might make them feel less satisfied, even if the treatment helps.

## 5. Conclusions

Psoriasis has a profound impact on patients’ lives, causing significant psychological burdens, including difficulties in interpersonal relationships, social interactions, and overall quality of life. In addition, patients often undergo a prolonged and frustrating journey of seeking medical treatment before receiving effective therapy. Here, we demonstrate a significant improvement in patients’ perception and cognition of body image and overall quality of life with the intervention of biologic agents. Our findings suggest that compassion and empathy play a crucial role in bridging the gap in treatment resistance, and healthcare professionals must find effective ways to enhance patients’ willingness to undergo treatment, promote their health, and alleviate their suffering.

## Figures and Tables

**Table 1:** Characteristics of the study participants (N = 44).

Sociodemographic Variables	n	%	Cumulative %
Gender			
Males	33	75.00	75.00
Females	11	25.00	100.00
Age			
40 years old and younger	9	20.45	20.45
41 to 50 years old	20	45.45	65.91
51 years old and older	15	34.09	100.00
Education level			
High school or below	22	50.00	50.00
College or above	22	50.00	100.00
Socioeconomic status *			
High	2	4.55	4.55
Middle to high	14	31.82	36.36
Middle	17	38.64	75.00
Middle to low	11	25.00	100.00
Marital status			
Single	11	25.00	25.00
Married	27	61.36	86.36
Other	6	13.64	100.00
Religion			
None	23	52.27	52.27
Buddhism	10	22.73	75.00
Taoism	11	25.00	100.00
Income			
<19,999 NTD	5	11.36	11.36
20,000–39,999 NTD	14	31.82	43.18
40,000–59,999 NTD	12	27.27	70.45
>60,000 NTD	13	29.55	100.00
Chronic diseases			
No	19	43.18	43.18
Yes	25	56.82	100.00
Major locations of psoriatic lesions			
Scalp	8	18.18	18.18
Face	8	18.18	36.36
Trunk	12	27.27	63.64
Limbs	16	36.36	100.00
BMI groups **			
Healthy body weight	11	25.00	25.00
Non-healthy body weight	33	75.00	100.00
Smoking			
Non-smoker	25	56.82	56.82
Current smoker	15	34.09	90.91
Former smoker	4	9.09	100.00
Alcohol consumption			
No	30	68.18	68.18
Yes	11	25.00	93.18
Quit	3	6.82	100.00
Regular exercise			
No	20	45.45	45.45
Yes	24	54.55	100.00
PASI severity			
Mild	10	22.73	22.73
Moderate to severe	34	77.27	100.00
Total sample size	44		

Note *: Socioeconomic status is classified into five levels based on educational background and occupational category: Level I—high socioeconomic status; Level II—middle-to-high socioeconomic status; Level III—middle socioeconomic status; Level IV—middle-to-low socioeconomic status; Level V—low socioeconomic status. Note **: BMI is divided into two groups. The non-healthy body weight group includes individuals with BMI < 18.5 and BMI ≥ 24. The healthy body weight group includes individuals with BMI > 18.5 and BMI < 24 (Health Promotion Administration, Ministry of Health and Welfare, 2018).

**Table 2:** Statistical analysis of sociodemographic variables and body image using the *t*-test (N = 44).

Sociodemographic Variables	Subgroup	N	Average	Standard Deviation	*t*-Value	*p*-Value
Gender	Male	33	18.21	6.19	−1.233	0.225
	Female	11	20.91	6.56		
Educational level	High school or below	22	21.95	4.41	3.652 **	0.001
	College or above	22	15.82	6.53		
Chronic diseases	No	19	17.42	6.34	−1.353	0.183
	Yes	25	20.00	6.20		
BMI groups	Healthy body weight	11	19.27	5.37	0.231	0.818
	Non-healthy body weight	33	18.76	6.68		
Regular exercise	No	20	18.15	6.68	−0.701	0.487
	Yes	24	19.50	6.08		
PASI severity	Mild	10	17.60	5.42	−0.728	0.471
	Moderate to severe	34	19.26	6.59		
Age	40 years old and younger	9	17.78	9.00	0.466 ^#^	0.631
	41 to 50 years old	20	18.45	5.83		
	51 years old and older	15	20.13	5.26		
Socioeconomic status	Middle to high	16	15.88	7.16	3.316 * ^# &^	0.046
	Middle	17	21.12	4.27		
	Middle to low	11	19.82	6.46		
Marital status	Single	11	18.09	5.56	1.931^#^	0.158
	Married	27	18.19	6.72		
	Other	6	23.50	4.18		
Religion	None	23	16.57	6.52	3.891^#^ * ^$^	0.028
	Buddhism	10	22.30	6.85		
	Taoism	11	20.64	2.94		
Income	<19,999 NTD	5	16.00	6.60	1.525 ^#^	0.223
	20,000–39,999 NTD	14	21.50	4.97		
	40,000–59,999 NTD	12	17.08	5.21		
	>60,000 NTD	13	18.85	7.89		
Major locations of psoriatic lesions	Scalp	8	17.50	3.46	0.271 ^#^	0.846
	Face	8	18.13	8.06		
	Trunk	12	19.92	5.57		
	Limbs	16	19.19	7.32		
Smoking	Non-smoker	25	18.72	5.93	0.620 ^#^	0.543
	Current smoker	15	19.93	6.03		
	Former smoker	4	16.00	10.23		
Alcohol consumption	No	30	17.90	6.58	1.298 ^#^	0.284
	Yes	11	21.45	4.32		
	Quit	3	19.33	9.29		

Note: * *p* < 0.05; ** *p* < 0.01, Note: ^#^ F-value, one-way analysis of variance (ANOVA) was used to explore the differences in the variable. Note: ^&^ No significant differences were found in post hoc comparisons using the Scheffé method. Note: ^$^ Post hoc comparisons using the Scheffé method revealed that Buddhist showed a higher level of impact on their body image compared to those who identified as non-religious.

**Table 3:** Statistical analysis of gender differences and quality of life using the *t*-test (N = 44).

Aspect	Gender	N	Average	Standard Deviation	*t*-Value	*p*-Value
Quality of Life	M	33	16.09	7.86	−0.603	0.550
	F	11	17.73	7.59		
Disease Symptoms	M	33	4.15	1.50	−0.777	0.441
	F	11	4.55	1.29		
Daily Activities	M	33	3.27	1.92	−0.416	0.680
	F	11	3.55	1.75		
Leisure Activities	M	33	3.09	1.89	0.562	0.577
	F	11	2.73	1.74		
Work and Study	M	33	1.33	1.19	−2.130 *	0.039
	F	11	2.18	0.98		
Interpersonal Relationships	M	33	2.15	1.86	−0.874	0.387
	F	11	2.73	2.00		
Treatment	M	33	2.09	0.95	0.259	0.797
	F	11	2.00	1.18		

Note: * *p* < 0.05

**Table 4:** Differences in mean scores of body image between pre- and post-tests (N = 44).

	Pre-Test		Post-Test		*t*-Value	*p*-Value
	(1)M	(2)SD	(1)M	(2)SD		
Body Image	18.89	6.32	4.93	4.09	14.205 ***	0.000

Note: *** *p* < 0.001; M = Mean, SD = Standard Deviation.

**Table 5:** Differences in mean scores of quality of life between pre- and post-tests (N = 44).

Aspect	Pre-Test		Post-Test		*t*-Value	*p*-Value
	(1)M	(2)SD	(1)M	(2)SD		
Disease Symptoms	4.25	0.98	1.45	1.07	13.408 ***	0.000
Daily Activities	3.34	0.75	1.87	0.97	7.956 ***	0.000
Leisure Activities	3.00	0.50	1.84	0.76	8.128 ***	0.000
Work and Study	1.55	0.27	1.19	0.62	6.404 ***	0.000
Interpersonal Relationships	2.30	0.34	1.89	0.68	6.681 ***	0.000
Treatment	2.07	0.36	1.00	0.49	11.286 ***	0.000
Quality of Life	16.50	3.20	7.74	3.64	10.556 ***	0.000

Note: *** *p* < 0.001; M = Mean, SD = Standard Deviation.

**Table 6:** Pre-test correlation analysis between body image and overall QoL (N = 44).

	Pre-Test Body Image
Pre-test quality of life Significance (two tailed)	0.726 ** 0.000

Note: ** Significant at the 0.01 level (two-tailed).

**Table 7:** Post-test correlation analysis between body image and overall QoL (N = 44).

	Post-Test Body Image
Post-test quality of life Significance (two tailed)	0.690 ** 0.000

Note: ** Significant at the 0.01 level (two tailed).

**Table 8:** Regression analysis of pre-test on body image and overall QoL (N = 44).

Variable	Non-Standardized Coefficient		Standardized Coefficient		T	*p*-Value
	B		Beta			
Body Image	0.888		0.726		6.841	0.000

Note: *R*^2^ = 0.527; Adjusted *R*^2^ = 0.516; Durbin-Watson = 1.657.

## Data Availability

The data that support the findings of this study are available from the corresponding authors upon reasonable request.

## Supplementary Materials

The following supporting information can be downloaded at: https://www.mdpi.com/article/10.3390/medicina60010160/s1, Table S1: Statistical analysis of education level and quality of life by *t*-test (N = 44); Table S2: Statistical analysis of chronic diseases and quality of life by *t*-test (N = 44); Table S3: Statistical analysis of BMI groups and quality of life by *t*-test (N = 44); Table S4: Statistical analysis of regular exercise and quality of life by *t*-test (N = 44); Table S5: Statistical analysis of PASI severity and quality of life by *t*-test (N = 44); Table S6: Statistical analysis of age and quality of life by *t*-test (N = 44); Table S7: Statistical analysis of socioeconomic status and quality of life by *t*-test (N = 44); Table S8: Statistical analysis of marital status and quality of life by *t*-test (N = 44); Table S9: Statistical analysis of religion and quality of life by *t*-test (N = 44); Table S10: Statistical analysis of income and quality of life by *t*-test (N = 44); Table S11: Statistical analysis of major locations of psoriasis lesions and quality of life by *t*-test (N = 44).; Table S12: Statistical analysis of smoking and quality of life by *t*-test (N = 44); Table S13: Statistical analysis of alcohol consumption and quality of life by *t*-test (N = 44).
