# Supplementary material for: Exploring the Effectiveness of Biological Therapy in Patients with Psoriasis: Body Image and Quality of Life
Source: Medicina (Kaunas). 2024 Jan 15;60(1):160. doi: 10.3390/medicina60010160 (PMC10819774; doi:10.3390/medicina60010160)
Supplement: Supplementary file 1 [file medicina-60-00160-s001.zip › medicina-2816759-supplementary.pdf]

Table S1. Statistical analysis of education level and quality of life by *t*-test (N = 44).

Aspect	Education Level	N	Average	Standard Deviation	*t*-Value	*p*-Value
Quality of Live	High school or below	22	20.55	5.65	4.040 ***	0.000
	College or above	22	12.45	7.50		
Disease Symptoms	High school or below	22	5.00	0.98	3.984 ***	0.000
	College or above	22	3.50	1.47		
Daily Activities	High school or below	22	4.32	1.49	4.045 ***	0.000
	College or above	22	2.36	1.71		
Leisure Activities	High school or below	22	3.82	1.59	3.258 **	0.002
	College or above	22	2.18	1.74		
Work and Study	High school or below	22	1.77	1.23	1.276	0.209
	College or above	22	1.32	1.13		
Interpersonal Relationships	High school or below	22	3.09	1.72	3.055 **	0.004
	College or above	22	1.50	1.74		
Treatment	High school or below	22	2.55	0.80	3.584 **	0.001
	College or above	22	1.59	0.96		

Note: ** $p < 0.01$; *** $p < 0.001$.

Table S2. Statistical analysis of chronic diseases and quality of life by *t*-test (N = 44).

Aspect	Chronic Diseases	N	Average	Standard Deviation	*t*-Value	*p*-Value
Quality of Live	No	19	15.37	7.14	−0.843	0.404
	Yes	25	17.36	8.20		
Disease Symptoms	No	19	4.11	1.10	−0.606	0.548
	Yes	25	4.36	1.68		
Daily Activities	No	19	3.00	1.83	−1.057	0.296
	Yes	25	3.60	1.89		
Leisure Activities	No	19	2.95	1.78	−0.163	0.871
	Yes	25	3.04	1.93		
Work and Study	No	19	1.63	1.16	0.414	0.681
	Yes	25	1.48	1.23		
Interpersonal Relationships	No	19	1.79	1.81	−1.577	0.122
	Yes	25	2.68	1.89		
Treatment	No	19	1.89	0.94	−1.006	0.320
	Yes	25	2.20	1.04		

Table S3. Statistical analysis of BMI groups and quality of life by *t*-test (N = 44).

Aspect	BMI Groups	N	Average	Standard Deviation	*t*-Value	*p*-Value
Quality of Live	Healthy body weight	11	15.18	6.48	0.231	0.818
	Non-healthy body weight	33	16.94	8.15		
Disease Symptoms	Healthy body weight	11	4.36	1.36	−0.648	0.520
	Non-healthy body weight	33	4.21	1.49		
Daily Activities	Healthy body weight	11	2.91	1.51	0.297	0.768

	Non-healthy body weight	33	3.48	1.97		
Leisure Activities	Healthy body weight	11	2.64	1.21	−0.884	0.382
	Non-healthy body weight	33	3.12	2.01		
Work and Study	Healthy body weight	11	1.18	.98	−0.752	0.456
	Non-healthy body weight	33	1.67	1.24		
Interpersonal Relationships	Healthy body weight	11	2.00	1.90	−1.175	0.247
	Non-healthy body weight	33	2.39	1.90		
Treatment	Healthy body weight	11	2.09	1.22	−0.595	0.555
	Non-healthy body weight	33	2.06	.93		

Table S4. Statistical analysis of regular exercise and quality of life by *t*-test (N = 44).

Aspect	Regular Exercise	N	Average	Standard Deviation	*t*-value	*p*-value
Quality of Live	No	20	15.85	7.18	−0.504	0.617
	Yes	24	17.04	8.28		
Disease Symptoms	No	20	4.15	1.57	−0.414	0.681
	Yes	24	4.33	1.37		
Daily Activities	No	20	3.25	1.83	−0.292	0.772
	Yes	24	3.42	1.93		
Leisure Activities	No	20	2.95	1.73	−0.162	0.872
	Yes	24	3.04	1.97		
Work and Study	No	20	1.25	1.12	−1.526	0.134
	Yes	24	1.79	1.22		
Interpersonal Relationships	No	20	2.15	1.73	−0.462	0.646
	Yes	24	2.42	2.04		
Treatment	No	20	2.10	0.97	0.191	0.849
	Yes	24	2.04	1.04		

Table S5. Statistical analysis of PASI severity and quality of life by *t*-test (N = 44).

Aspect	PASI Severity	N	Average	Standard Deviation	*t*-Value	*p*-Value
Quality of Live	Mild	10	12.90	7.68	−1.711	0.094
	Moderate to Severe	34	17.56	7.54		
Disease Symptoms	Mild	10	3.60	1.71	−1.646	0.107
	Moderate to Severe	34	4.44	1.33		
Daily Activities	Mild	10	2.50	1.72	−1.653	0.106
	Moderate to Severe	34	3.59	1.86		
Leisure Activities	Mild	10	2.00	1.56	−2.021	0.050
	Moderate to Severe	34	3.29	1.83		
Work and Study	Mild	10	1.40	1.17	−0.435	0.665
	Moderate to Severe	34	1.59	1.21		
Interpersonal Relationships	Mild	10	1.90	1.97	−0.750	0.457
	Moderate to Severe	34	2.41	1.88		
Treatment	Mild	10	1.50	1.08	−2.132 *	0.039
	Moderate to Severe	34	2.24	0.92		

Table S6. Statistical analysis of age and quality of life by *t*-test (N = 44).

Aspect	Age	N	Average	Standard Deviation	*F*-Value	*p*-Value	Scheffé Post hoc Comparison
Quality of Live	40 years old and below	9	17.22	8.44	0.049	0.953	NA
	41 to 50 years old	20	16.25	7.60			
	51 years old and above	15	16.40	8.02			
Disease Symptoms	40 years old and below	9	4.44	1.51	0.124	0.883	NA
	41 to 50 years old	20	4.15	1.42			
	51 years old and above	15	4.27	1.53			
Daily Activities	40 years old and below	9	3.44	2.01	0.064	0.938	NA
	41 to 50 years old	20	3.40	1.82			
	51 years old and above	15	3.20	1.97			
Leisure Activities	40 years old and below	9	3.89	1.83	1.341	0.273	NA
	41 to 50 years old	20	2.75	1.68			
	51 years old and above	15	2.80	2.01			
Work and Study	40 years old and below	9	1.33	1.12	0.539	0.588	NA
	41 to 50 years old	20	1.75	1.29			
	51 years old and above	15	1.40	1.12			
Interpersonal Relationships	40 years old and below	9	2.11	2.26	0.817	0.449	NA
	41 to 50 years old	20	2.00	1.45			
	51 years old and above	15	2.80	2.18			
Treatment	40 years old and below	9	2.00	1.12	0.322	0.726	NA
	41 to 50 years old	20	2.20	0.83			
	51 years old and above	15	1.93	1.16			

comparison; n.s. indicates no significant difference after post hoc comparison.

Table S7. Statistical analysis of socioeconomic status and quality of life by *t*-test (N = 44).

Aspect	Socioeconomic Status	N	Average	Standard Deviation	*F*-value	*p*-value	Scheffé Post hoc Comparison
Quality of Live	(1) Middle-to-high	16	12.69	8.72	3.648 *	0.035	(2) > (1)
	(2) Middle	17	19.41	6.22			
	(3) Middle-to-low	11	17.55	6.53			
Disease Symptoms	(1) Middle-to-high	16	3.44	1.67	4.621 *	0.015	(2) > (1)
	(2) Middle	17	4.71	.85			
	(3) Middle-to-low	11	4.73	1.42			
Daily Activities	(1) Middle-to-high	16	2.50	1.90	2.869	0.068	NA
	(2) Middle	17	3.94	1.75			
	(3) Middle-to-low	11	3.64	1.69			
Leisure Activities	(1) Middle-to-high	16	2.31	1.92	2.095	0.136	NA
	(2) Middle	17	3.59	1.70			
	(3) Middle-to-low	11	3.09	1.76			
Work and Study	(1) Middle-to-high	16	1.38	1.20	1.667	0.201	NA
	(2) Middle	17	1.94	1.20			
	(3) Middle-to-low	11	1.18	1.08			
Interpersonal Relationships	(1) Middle-to-high	16	1.38	1.86	3.564 *	0.037	(2) > (1)
	(2) Middle	17	3.00	1.62			
	(3) Middle-to-low	11	2.55	1.92			
Treatment	(1) Middle-to-high	16	1.69	0.95	1.971	0.152	NA
	(2) Middle	17	2.24	0.83			

Aspect	Socioeconomic Status	N	Average	Standard Deviation	*F*-value	*p*-value	Scheffé Post hoc Comparison
	(3) Middle-to-low	11	2.36	1.21			

NA indicates no need for post hoc comparison; n.s. indicates no significant difference after post hoc comparison.

Table S8. Statistical analysis of marital status and quality of life by *t*-test (N = 44).

Aspect	Marital Status	N	Average	Standard Deviation	*F*-Value	*p*-Value	Scheffé Post hoc Comparison
Quality of Live	(1) Single	11	16.55	6.38	0.027	0.973	NA
	(2) Married	27	16.33	8.52			
	(3) Others	6	17.17	7.41			
Disease Symptoms	(1) Single	11	4.45	1.37	0.179	0.837	NA
	(2) Married	27	4.15	1.56			
	(3) Others	6	4.33	1.21			
Daily Activities	(1) Single	11	3.18	1.47	0.062	0.940	NA
	(2) Married	27	3.37	2.06			
	(3) Others	6	3.50	1.87			
Leisure Activities	(1) Single	11	3.36	1.50	0.292	0.749	NA
	(2) Married	27	2.85	2.01			
	(3) Others	6	3.00	1.79			
Work and Study	(1) Single	11	1.45	1.13	0.188	0.830	NA
	(2) Married	27	1.63	1.21			
	(3) Others	6	1.33	1.37			
Interpersonal Relationships	(1) Single	11	2.00	1.48	0.546	0.583	NA
	(2) Married	27	2.26	1.97			
	(3) Others	6	3.00	2.28			
Treatment	(1) Single	11	2.09	1.04	0.017	0.984	NA
	(2) Married	27	2.07	0.96			
	(3) Others	6	2.00	1.26			

NA indicates no need for post hoc comparison; n.s. indicates no significant difference after post hoc comparison.

Table S9. Statistical analysis of religion and quality of life by *t*-test (N = 44).

Aspect	Religion	N	Average	Standard Deviation	*F*-value	*p*-value	Scheffé Post hoc Comparison
Quality of Live	(1) None	23	15.35	7.00	2.345	0.109	NA
	(2) Buddhism	10	21.00	8.71			
	(3) Taoism	11	14.82	7.40			
Disease Symptoms	(1) None	23	4.22	1.35	0.450	0.641	NA
	(2) Buddhism	10	4.60	1.35			
	(3) Taoism	11	4.00	1.79			
Daily Activities	(1) None	23	3.17	1.90	0.806	0.453	NA
	(2) Buddhism	10	4.00	2.00			
	(3) Taoism	11	3.09	1.70			
Leisure Activities	(1) None	23	2.78	1.83	2.605	0.086	NA
	(2) Buddhism	10	4.10	1.85			
	(3) Taoism	11	2.45	1.57			

Aspect	Religion	N	Average	Standard Deviation	*F*-value	*p*-value	Scheffé Post hoc Comparison
Work and Study	(1) None	23	1.48	1.12	1.688	0.197	NA
	(2) Buddhism	10	2.10	1.20			
	(3) Taoism	11	1.18	1.25			
Interpersonal Relationships	(1) None	23	1.70	1.49	5.956 **	0.005	(2) > (1)
	(2) Buddhism	10	3.90	2.08			
	(3) Taoism	11	2.09	1.76			
Treatment	(1) None	23	2.00	0.90	0.339	0.715	NA
	(2) Buddhism	10	2.30	1.25			
	(3) Taoism	11	2.00	1.00			

NA indicates no need for post hoc comparison; n.s. indicates no significant difference after post hoc comparison.

Table S10. Statistical analysis of income and quality of life by t-test (N = 44).

Aspect	Income	N	Average	Standard Deviation	*F*-Value	*p*-Value	Scheffé Post hoc Comparison
Quality of Live	(1) < 19,999 NTD	5	15.60	6.80	0.750	0.529	NA
	(2)20,000–39,999 NTD	14	19.07	6.93			
	(3)40,000–59,999 NTD	12	15.42	8.11			
	(4) > 60,000 NTD	13	15.08	8.67			
Disease Symptoms	(1) <19,999 NTD	5	3.80	1.10	1.764	0.169	NA
	(2)20,000–39,999元	14	4.93	1.07			
	(3)40,000–59,999元	12	4.17	1.80			
	(4)60,000元以上	13	3.77	1.42			
Daily Activities	(1) < 19,999 NTD	5	3.60	1.95	0.593	0.623	NA
	(2) 20,000–39,999 NTD	14	3.79	1.97			
	(3) 40,000–59,999 NTD	12	3.25	1.86			
	(4) > 60,000 NTD	13	2.85	1.82			
Leisure Activities	(1) < 19,999 NTD	5	2.40	1.82	0.751	0.528	NA
	(2) 20,000–39,999 NTD	14	3.57	1.91			
	(3) 40,000–59,999 NTD	12	2.67	1.87			
	(4) > 60,000 NTD	13	2.92	1.80			
Work and Study	(1) < 19,999 NTD	5	1.40	1.52	0.111	0.953	NA
	(2) 20,000–39,999 NTD	14	1.64	1.15			
	(3) 40,000–59,999 NTD	12	1.42	1.16			
	(4) > 60,000 NTD	13	1.62	1.26			
Interpersonal Relationships	(1) < 19,999 NTD	5	2.20	2.28	0.620	0.606	NA
	(2) 20,000–39,999 NTD	14	2.86	1.70			
	(3) 40,000–59,999 NTD	12	1.92	1.62			
	(4) > 60,000 NTD	13	2.08	2.22			
Treatment	(1) < 19,999 NTD	5	2.20	1.30	0.466	0.707	NA
	(2) 20,000–39,999 NTD	14	2.29	1.07			
	(3) 40,000–59,999 NTD	12	2.00	1.04			
	(4) > 60,000 NTD	13	1.85	0.80			

NA indicates no need for post hoc comparison; n.s. indicates no significant difference after post hoc comparison.

Table S11. Statistical analysis of major locations of psoriasis lesions and quality of life by *t*-test (N = 44).

Aspect	Major Locations of Psoriasis Lesions	N	Average	Standard Deviation	*F*-Value	*p*-Value	Scheffé Post hoc Comparison
Quality of Live	(1) Scalp	8	13.88	6.03	1.842	0.155	NA
	(2) Face	8	12.75	9.62			
	(3) Trunk	12	16.75	7.88			
	(4) Limbs	16	19.50	6.71			
Disease Symptoms	(1) Scalp	8	4.00	1.20	1.323	0.280	NA
	(2) Face	8	3.50	2.14			
	(3) Trunk	12	4.33	1.07			
	(4) Limbs	16	4.69	1.35			
Daily Activities	(1) Scalp	8	2.75	1.75	1.681	0.186	NA
	(2) Face	8	2.50	2.07			
	(3) Trunk	12	3.33	1.97			
	(4) Limbs	16	4.06	1.61			
Leisure Activities	(1) Scalp	8	2.38	2.13	1.593	0.206	NA
	(2) Face	8	2.38	2.07			
	(3) Trunk	12	2.83	2.04			
		16	3.75	1.24			
Work and Study	(1) Scalp	8	0.88	0.99	3.262 *	0.031	n.s
	(2) Face	8	0.88	0.99			
	(3) Trunk	12	1.75	1.22			
	(4) Limbs	16	2.06	1.12			
Interpersonal Relationships	(1) Scalp	8	2.00	2.00	0.124	0.946	NA
	(2) Face	8	2.25	2.43			
	(3) Trunk	12	2.25	1.96			
	(4) Limbs	16	2.50	1.63			
Treatment	(1) Scalp	8	1.88	0.83	3.176 *	0.034	(4) > (2)
	(2) Face	8	1.25	1.28			
	(3) Trunk	12	2.25	0.87			
	(4) Limbs	16	2.44	0.81			

NA indicates no need for post hoc comparison; n.s. indicates no significant difference after post hoc comparison.

Table S12. Statistical analysis of smoking and quality of life by *t*-test (N = 44).

Aspect	Smoking	N	Average	Standard Deviation	*F*-value	*p*-Value	Scheffé Post hoc Comparison
Quality of Live	(1) Non-smoker	25	15.36	7.78	0.660	0.522	NA
	(2) Current smoker	15	17.73	7.91			
	(3) Former smoker	4	19.00	7.39			
Disease Symptoms	(1) Non-smoker	25	4.16	1.60	0.266	0.768	NA
	(2) Current smoker	15	4.47	1.25			
	(3) Former smoker	4	4.00	1.41			
Daily Activities	(1) Non-smoker	25	2.96	1.81	1.347	0.271	NA
	(2) Current smoker	15	3.73	1.83			
	(3) Former smoker	4	4.25	2.22			
Leisure Activities	(1) Non-smoker	25	2.60	1.78	1.572	0.220	NA
	(2) Current smoker	15	3.40	1.96			
	(3) Former smoker	4	4.00	1.41			

Aspect	Smoking	N	Average	Standard Deviation	*F*-value	*p*-Value	Scheffé Post hoc Comparison
Work and Study	(1) Non-smoker	25	1.52	1.16	0.320	0.728	NA
	(2) Current smoker	15	1.47	1.25			
	(3) Former smoker	4	2.00	1.41			
Interpersonal Relationships	(1) Non-smoker	25	2.24	1.81	0.033	0.967	NA
	(2) Current smoker	15	2.40	2.13			
	(3) Former smoker	4	2.25	1.89			
Treatment	(1) Non-smoker	25	1.88	1.09	1.123	0.335	NA
	(2) Current smoker	15	2.27	.80			
	(3) Former smoker	4	2.50	1.00			

NA indicates no need for post hoc comparison; n.s. indicates no significant difference after post hoc comparison.

Table S13. Statistical analysis of alcohol consumption and quality of life by *t*-test (N = 44).

Aspect	Alcohol Consumption	N	Average	Standard Deviation	*F*-Value	*p*-Value	Scheffé Post hoc comparison
Quality of Live	(1) No	30	16.07	7.66	0.152	0.859	NA
	(2) Yes	11	17.27	7.52			
	(3) Quitted	3	18.00	12.00			
Disease Symptoms	(1) No	30	4.20	1.54	0.054	0.947	NA
	(2) Yes	11	4.36	1.29			
	(3) Quitted	3	4.33	1.53			
Daily Activities	(1) No	30	3.27	1.89	0.204	0.817	NA
	(2) Yes	11	3.36	1.75			
	(3) Quitted	3	4.00	2.65			
Leisure Activities	(1) No	30	2.87	1.85	.239	0.788	NA
	(2) Yes	11	3.27	1.62			
	(3) Quitted	3	3.33	3.06			
Work and Study	(1) No	30	1.70	1.15	0.851	0.434	NA
	(2) Yes	11	1.27	1.19			
	(3) Quitted	3	1.00	1.73			
Interpersonal Relationships	(1) No	30	2.07	1.68	0.690	0.507	NA
	(2) Yes	11	2.82	2.18			
	(3) Quitted	3	2.67	3.06			
Treatment	(1) No	30	1.97	1.07	0.758	0.475	NA
	(2) Yes	11	2.18	0.87			
	(3) Quitted	3	2.67	0.58			

NA indicates no need for post hoc comparison; n.s. indicates no significant difference after post hoc comparison.
